# Predicting and Interpreting the Structure of Type IV Pilus of Electricigens by Molecular Dynamics Simulations

**DOI:** 10.3390/molecules22081342

**Published:** 2017-08-12

**Authors:** Chuanjun Shu, Ke Xiao, Changchang Cao, Dewu Ding, Xiao Sun

**Affiliations:** State Key Laboratory of Bioelectronics, School of Biological Science and Medical Engineering, Southeast University, Nanjing 210096, China; chuanjunshu@seu.edu.cn (C.S.); kxiao@seu.edu.cn (K.X.); caochch@gmail.com (C.C.); dwding2008@aliyun.com (D.D.)

**Keywords:** electron transfer, electricigen, *Geobacter*, molecular dynamics simulation, symmetric docking

## Abstract

Nanowires that transfer electrons to extracellular acceptors are important in organic matter degradation and nutrient cycling in the environment. *Geobacter* pili of the group of Type IV pilus are regarded as nanowire-like biological structures. However, determination of the structure of pili remains challenging due to the insolubility of monomers, presence of surface appendages, heterogeneity of the assembly, and low-resolution of electron microscopy techniques. Our previous study provided a method to predict structures for Type IV pili. In this work, we improved on our previous method using molecular dynamics simulations to optimize structures of *Neisseria gonorrhoeae* (GC), *Neisseria meningitidis* and *Geobacter uraniireducens* pilus. Comparison between the predicted structures for GC and *Neisseria meningitidis* pilus and their native structures revealed that proposed method could predict Type IV pilus successfully. According to the predicted structures, the structural basis for conductivity in *G.uraniireducens* pili was attributed to the three N-terminal aromatic amino acids. The aromatics were interspersed within the regions of charged amino acids, which may influence the configuration of the aromatic contacts and the rate of electron transfer. These results will supplement experimental research into the mechanism of long-rang electron transport along pili of electricigens.

## 1. Introduction

Electricigens that can transfer electrons extracellularly participate widely in geochemical cycling of metals, minerals, and carbon in the environment. These microorganisms have been applied to bioremediation of contaminated environments and studied in microbe–electrode interactions [1]. Among these electricigens, the *Geobacter* species produce electrically conductive pili that are thought to play a critical role in long-range electron transfer to extracellular Fe(III) oxides and other electron acceptors [2,3]. These bio-electronic materials, which belong to Type IV pili (T4P), are attractive because they can be obtained from sustainable feedstocks without the need for toxic solvents or harsh chemical processes. This means that potentially toxic components can be avoided in the final products [4].

The *Geobacter* pili may be regarded as “microbial nanowires” because of their electrical conductivity [2,3,5]. These pili have a common origin and similar structure to Type II secretion systems and Type IV pili [6,7,8]. These systems use conserved ATP-powered machinery to assemble helical fibers, which are composed of thousands of identical monomers (so-called pilins) [9,10,11]. Pre-pilin is cleaved off at a conservative peptidase splice site to form a mature pilin [12]. Then, a new N-terminal is methylated and the monomers assemble into a pilus [13,14,15].

The monomers of T4Ps have a distinct N-terminal signal sequence and a highly conservative hydrophobic spiral, which form a conservative structural domain in protein–protein interactions and specific DNA recognition [16,17]. Downstream sequences of this hallmark sequence have a high variability and the total length of pilins varies from 90 to more than 150 amino acids [18,19,20]. The diversity of pilins results in different assembly modes and a diverse range of cellular functions including surface motility, biofilm formation, and electron transport [2,11,20,21,22]. The latter is important for the characteristics and behavior of electricigens such as *G. sulfurreducens* and *G. uraniireducens*. For example, the conductivity of *G. sulfurreducens* pilus is attributed to a truncated pilin (61 amino acids), which is substantially shorter than normal pilin found in *G. uraniireducens* (193 amino acids). The conductivity of the latter is 3 × 10^−4^ S/cm, which is much lower than that of the former at the same pH (5 × 10^−2^ S/cm) [5]. Understanding the structure of these monomers and their assembled conformation is important for comprehending their functions, and in particular their roles in the process of electron transport [1].

To date X-ray crystal structural analysis and nuclear magnetic resonance (NMR) have been used to construct full atomic structures of proteins; however, these approaches face difficulties due to the insolubility of the monomers, the presence of surface appendages, and the heterogeneity of the whole assembly for pilus [20]. These difficulties for X-ray and NMR are also challenges for the cryo-electron microscopy. Although structures can be obtained by cryo-electron microscopy (Cryo-EM), this technique is unaffordable for many investigators and conflicting atomic positions exist in the constructed structures. Furthermore, Cryo-EM can only acquire 2D images of pilus [23]. The process of reconstruction of three-dimensional structures from electron micrographs is necessary [23,24]. Two typical intact assembled structures of T4Ps are the *Neisseria gonorrhoeae* (GC) and *Neisseria meningitidis* pilus, which have been resolved with a combination of X-ray crystallography and Cryo-EM (PDB ID: 2HIL and 5KUA), including computational docking of pilin structure into the reconstructed 3D image from Cryo-EM [11,20,25].

Owing to the fact that the known structures of helical fibers assemble symmetrically, previous methods have assumed that the monomers are rigid-bodies, which ignored the flexibility of the backbones [1,26]. Additionally, conflicting atomic positions may result from such homology modeling processes. In our previous study [27], an approach was developed to predict the full atomic models of pilus with symmetric docking and sparse constraints based on Rosetta, which weakened the assumption that of rigid-body monomers by a final step of backbone relaxation. However, the modeling method did not consider the side-chain flexibility [28,29].

Flexibility is a critical factor for the structural stability of proteins and their biological functions [30]. Pilins are flexible entities, and the pilin–pilin recognition process itself suggests structural rearrangements [6,31]. Thus, conformation adjustment is part of the assembly process from a structure point of view, but also in terms of energetics. Meanwhile, consideration of dynamic effects by docking algorithms is not yet routine; however, molecular dynamics (MD) simulations can be used to improve docking processes [31]. Additionally, explicit water MD simulations can be used to refine structural models to improve geometry and structural statistics [32]. Therefore, to improve on our previous approach, in this study, we applied MD simulations to form a more reasonable approach to predict the structure of these biological nanowires and interpret the details of their optimized conformations.

To verify the feasibility of the proposed method, *Neisseria gonorrhoeae* (GC) [11] and *Neisseria meningitidis* [25] pilus with known structures were remodeled and systematically investigated. The proposed method was then used to explore the structural basis for the conductivity of the *G. uraniireducens* pili, as an instance of the electricigen T4P. Previous studies indicated that the mechanism for *Geobacter* pilus to transfer electron along pili attributed, at least in part, to the conjugation of aromatic amino acids [4,33]. Therefore, the arrangement of aromatic amino acids is highlighted when we interpret the mechanism of long-distance electron transport in *G. uraniireducens* pili.

## 2. Results and Discussion

### 2.1. Predicted GC Pilus Was Closer to the Native Structure Than Previous Model

To improve on our previous approach [27], we used MD simulations to guide our predictions of the structure of T4Ps. The structure of *Neisseria gonorrhoeae* (GC) pilus, which is a known intact T4P structure, was reconstructed to validate the proposed method [11]. The GC monomer was obtained from the PDB database (PDB ID: 2HI2) [11].

Because of the drawbacks such as the static snapshot problem and signal interference, experimentally determined structures may have atomic conflicts where further refinement is needed [34]. Furthermore, as the NMR/X-ray-derived pilin structure was always derived from pilin monomers immersed in lipid micelles, we refined its structure in solution in MD simulations. Meanwhile, the accuracy of computational methods, no matter the homology modeling or the ab initio calculations, depends greatly on the aligned templates and the length of protein sequences, and could be further improved by refinement to high resolution [35]. Thus, we used MD simulations to refine the monomer structure, to improve the geometry and structural statistics [32]. One Na^+^ ion was added to neutralize the simulated system and we used 30 ns MD simulations with a PMEMD model in Amber12 software (TRII-Biotech, Shanghai, China) (Supplementary Materials Figure S1). When the MD simulation was completed, the frame with the lowest energy was selected, by the *cpptraj* model, as the refined monomer conformation for the symmetric docking in the next step. The root-mean-square deviation (RMSD) between the refined monomer and the X-ray structure (PDB ID:2HI2) was 0.761 Å, as calculated by PyMOL software(www.pymol.org), which was considered reasonable for further assembly.

**Figure 1:**
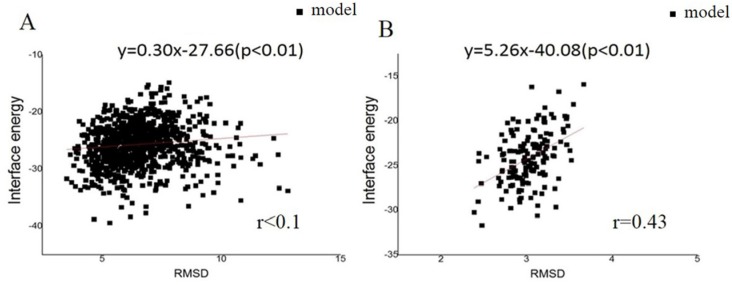
The landscape of interface energy score for the GC pili. The black dots stand for the distribution of RMSDs of the models. The red lines and formulae indicate the correlation between RMSD and energy. The results from: (**A**) the whole model set; and (**B**) the largest cluster.

To predict the initial pilus structure by symmetric docking, details of the helical symmetry parameters were set as follows: (1) Rise (Å) was set to be 5–15; (2) the rotation_angle (°) was set to be 80–130; (3) the radius_to_com (Å) was set to be 15–30; and (4) the number of monomers was set to be 15 (Figure S2). Pairs of residues F1/E5, V9/L16, R30/E49, K76/D153, K74/E113, and N99/R112 (an amino acid is represented by a single letter) were set as constrained pairs. These sparse constraints were utilized to narrow down the sampling space. After 1000 models were obtained from the Rosetta symmetric docking module, a clustering process (Cut-off = 1.75 Å) and energy scores were used to evaluate these models. The RMSDs were calculated by comparing models to the native structure (PDB: 2HIL). This comparison showed a tendency for models with larger RMSD appearing to have higher energy values (Figure 1). Models were discarded when the corresponding RMSDs were larger than the sum of the X-ray resolution and Cryo-EM resolution of 2.3 Å and 12.5 Å, respectively. From the RMSD data, good convergence of 1000 low-resolution models was found, and the structural energy score (total energy) positively correlated with the RMSD values. Such good convergence suggested a basin of low-energy conformations, within which the native structure tends to situate, to fold efficiently and retain robustness. This correlation was found regardless of which models were considered (Figure S3). Then, three input files (monomer, helical symmetry information, and the sparse constraint data) from the low-resolution phase were executed in the high-resolution phase, and 1000 high-resolution pilus models were obtained. After a clustering process based on RMSDs (Cutoff = 1.75 Å), the 1000 high-resolution models were divided into 93 clusters. The largest cluster had 150 models, and each of the remaining clusters contained less than 109 models. The interface energy was calculated as the difference between the total energy of the complex and the total energy when the partners are separated, as a function of RMSD, for all the models. The interface energy score was utilized because it can approximate binding energy and reflects the stability of pilin assembly. From the high-resolution models, good convergence phenomenon, and a positive correlation between the structural energy score (interface energy) and RMSD values could also be observed. Again, the correlation was found regardless of whether all models (929 models, Figure 1A, *r* < 0.1) or only those models in the largest cluster were considered (150 models, Figure 1B, *r* = 0.43). The result indicated that the predicted structures with lower energy always have smaller RMSDs, when aligned to the intact structure determined by cryo-EM. We chose an initial structure for further optimization according to the positive correlation.

Atomic positions may conflict and the values for bond lengths, bond angles, and dihedral angles may deviate from their true values in the assembled models because the monomers were handled as rigid-bodies during the symmetric docking [36]. Thus, MD simulations were performed to optimize the structure. The lowest energy model in the largest cluster from the last step, which consisted of 15 monomers, was chosen as the initial structure for MD simulation of the pilus. The chain names of these 15 monomers are denoted by consecutive letters: A to O. To decrease the size of the system, a short pilus fragment was extracted from the lowest energy model, which consisted of eight consecutive monomers (chain A to H, RMSD to native structure GC1 = 2.739). Eight Na^+^ ions were added to neutralize the simulated system. Then a 60 ns MD simulation was implemented with a PMEMD model in Amber12 software (Figure S4). The frame with the lowest energy from the MD simulation was chosen as the optimized structure with a *cpptraj* model in Amber12 software. The similarity between the Cryo-EM model and the optimized snapshot was good (Table 1), with an RMSD of 2.308 Å, which is within the X-ray crystallography resolution. As shown in Table 1, the optimized model had a lower RMSD value, when compared with that of the polymer models obtained by our previous method. Furthermore, to further verify the feasibility of the proposed method, the Type IV pilus of *Neisseria meningitidis* was also remodeled. The similarity between the Cryo-EM model (PDB ID:5KUA) and the optimized snapshot indicated that the optimized structure of *Neisseria meningitidis* pilus has a lower RMSD value (1.972 Å), compared to the result of previously published method (Table S1). Thus, we conclude that the monomer relaxation and optimization of the assembled pilus by MD simulation, yielded models that were more similar to the native structure.

GC1, GC2, GC3 and GC4 represent native structure (PDB ID:2HIL), pilus structure obtained from symmetric docking refined monomer, pilus structure obtained from previous method [27], and the optimized pilus structure, respectively. Data shown in the table were obtained by quantifying the conformational changes between the two structures (units are Å).

### 2.2. Structure of the G. Uraniireducens Pilus

The conductivity of *G. uraniireducens* pili has been reported to be 3 × 10^−4^ S/cm [5]. To investigate the structural basis for this conductivity, *G. uraniireducens* pilus was modeled with our proposed method.

#### 2.2.1. Monomer Structure of the *G. uraniireducens* Pilus

**Figure 2:**
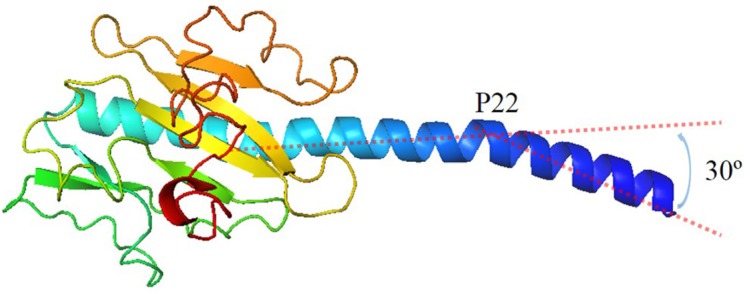
Structure of monomer in *G.uraniireducens* pilus. The bending of N-terminal helix is 30°. The position of curvature is the 22th amino acid.

Because the structure of *G. uraniireducens* pilin has not been experimentally determined [5,37], the conformation of the pilin was predicted using I-TASSER server (Figure 2) [38]. The threading templates used by I-TASSER were 2hi2A, 3sokA, 1oqwA, 2m7gA and 1ay2A. The bending of the N-terminal helix (30°) and the position of curvature (P22) for the *G. uraniireducens* pilin are as same as those of the GC monomer. The predicted conformation exhibited common structural features of full-length pilins: alpha-helix (1–51 amino acids) and consecutive anti-parallel beta-sheets (52–193 amino acids) [39]. The consecutive anti-parallel beta-sheets formed a globular head domain to protect the hydrophobic termini of the alpha-helix. Furthermore, there were 16 aromatic amino acids and 23 charged amino acids in the *G. uraniireducens* pilin, which likely play critical roles in the function and stability of *G. uraniireducens* pili.

#### 2.2.2. Predicted Structure of *G. uraniireducens* Pilus

**Figure 3:**
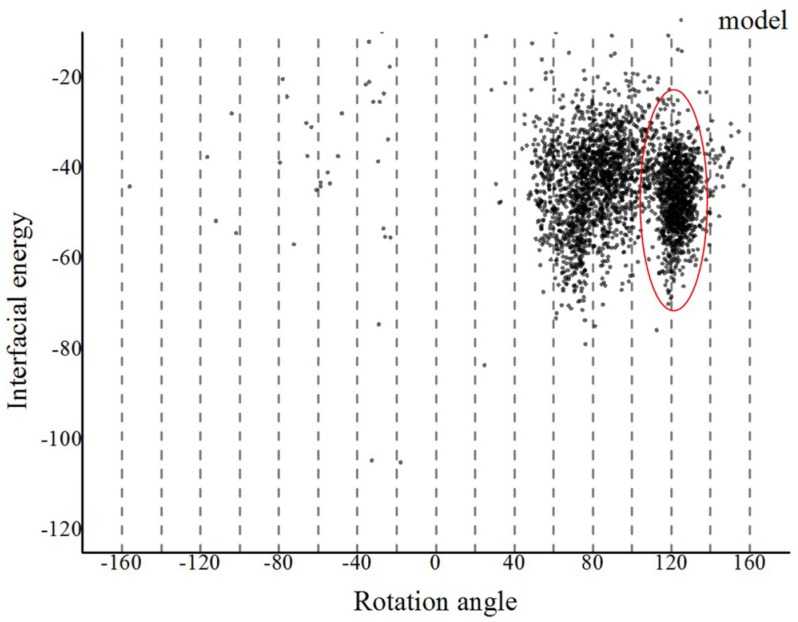
The landscape of interface energy score for the *G. uraniireducens* pili in the low-resolution phase. The interface energy is calculated as a function of rotation angle for all the models. Models tended to converge into one local trough with a rotation angle in the range 100–140°, as highlighted by the red ellipse.

Based on our proposed method, we first refined the predicted monomer of *G. uraniireducens* pilus via a 30 ns MD simulation (Figure S5). Three Na^+^ ions were added to neutralize the simulated system. The frame with the lowest energy from the MD simulation was selected as the refined monomer conformation using the *cpptraj* model in Amber12 software (Figure S6). Assembled models of *G. uraniireducens* pilus were then achieved with the symmetric docking module in Rosetta with sparse constraints [27,40,41]. Helical symmetry was inferred from a homologous experimentally determined structure of the GC pilus. Details of helical symmetry parameters for the low-resolution phase were set as follows: (1) Rise (Å) was set to be 5–15; (2) rotation_angle (°) was set to be −180–+180; (3) radius_to_com (Å) was set to be 15–30; and (4) the number of monomers was set to be 15. The aromatic amino acids and charged amino acids were set to be constrained pairs. Especially, because of the polar interactions between the positively charged N-terminal F1 on the terminal pilin subunit in the growing filament and the negatively charged E5 of the incoming subunit, the amino acid F1 and E5, were set as a constrained pair [11]. Then, we obtained 3000 models. As shown in Figure 3, these models converged into a single local trough, which was located at 100–140°. The low-energy region was taken as the starting range for the high-resolution phase.

**Figure 4:**
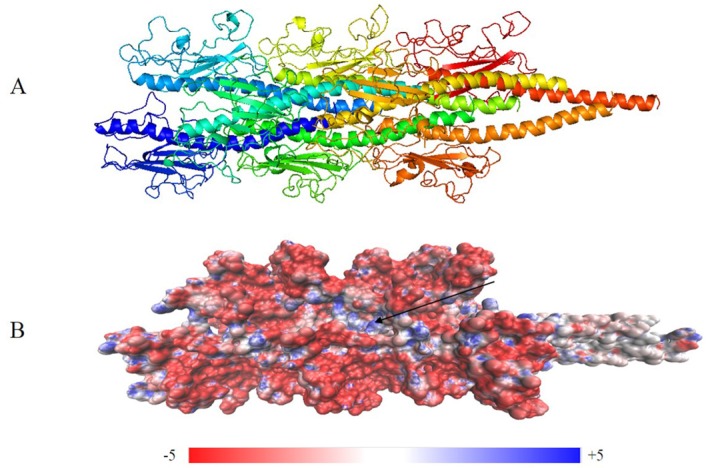
Optimized structure of the *G. uraniireducens* pilus: (**A**) filament structure containing eight monomers, each indicated by a different color; and (**B**) electrostatic potential distribution on the surface of the optimized structure. Red and blue represent negative and positive potential values, respectively. A deep groove is a possible extended DNA binding site (arrow).

In the high-resolution phase, 1000 models were obtained. A clustering process (Cut-off = 4 Å) and the interface energy score were used to evaluate the models. These 1000 high-resolution models were divided into 100 clusters. The largest cluster had 277 models, and each of the remaining clusters contained less than 134 models. The model with the lowest energy in the largest cluster was chosen for the next step (Figure S7). A short pilus fragment, which consisted of eight consecutive monomers, was extracted and optimized with the steepest descent algorithm (50,000 steps) and conjugate gradient algorithm (20,000 steps). Twenty-four Na+ ions were added to neutralize the simulated system. The optimized structure of the *G. uraniireducens* pilus is shown in Figure 4A. The subunits_per_turn, rise, rotation angle, and diameter of the pilus were 3, 14 Å, 116° and 69 Å, respectively. Similar details were shown by the other 14 lowest energy models in the largest cluster (Figure S8).

As shown in Figure 4, the surface of the optimized structure consisted of globular head domains of monomers. The electrostatic potential values of the pilus indicated that positive and negative potentials were mainly distributed in C-terminal spheres (Figure 4B). These characteristics suggest that C-terminal spheres are required for pilin folding and/or pilus assembly and are involved in pilus–pilus interactions. Furthermore, the electrostatic potential can be used to predict binding sites between the pilus and surface appendages. For example, a positively charged groove (Figure 4B) was wide enough to bind the negatively charged backbone of double-stranded DNA [42]. The charged, corrugated surface indicates sites where various binding events to pili may occur, including adherence to neighboring pilus for construction of the biofilm.

### 2.3. Potential Structural Basis for Conductivity in *G. uraniireducens* Pili

Energy flow in biological structures always requires submillisecond charge transport over long molecular distances [43]. Electron transport over long distances (>25 Å) likely involves multistep tunneling, often called hopping, in which redox-active amino acid side chains act as intermediate donors or acceptors [44]. Aromatic amino acid is one kind of amino acid which has the redox-active side chain. These aromatic amino acids are able to absorb energy which excites its electron to the excited state. Therefore, aromatic amino acid can facilitate electron transfer between distant redox centers. The arrangement of aromatic amino acids are highlighted when we explore the potential structural basis for the conductivity in *G. uraniireducens* pilus.

Genetic substitution of an alanine for each of the three tyrosines (+27, +32 and +57) and two of the phenylalanines (+24 and +51) in *Geobacter sulfurreducens* PilA, the pilus subunit, assembled pili with poor conductivity, suggesting the importance of aromatic amino acids in electron transport [33]. Meanwhile, there is no tryptophan which facilitates electron transport more effectively than tyrosines or phenylalanines in *Geobacter sulfurreducens* PilA, and previous study indicated that the conductivity of nanowires was increased when genetic substitution of a tryptophan for each of the two aromatic amino acids (+51 and +57) in *Geobacter sulfurreducens* PilA [4]. Furthermore, pilins of the *Geobacter sulfurreducens* and the *Geobacter uraniireducens* are homologous proteins. Hence, the mechanism for electron transfer along pili in *Geobacter uraniireducens* can also partially attributed to the conjugation of aromatic amino acids.

**Figure 5:**
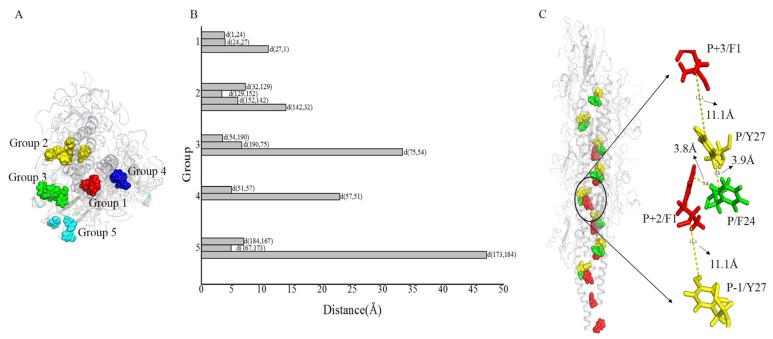
Details of the structure for *G. uraniireducens* pilus. The pilus structure is represented schematically in transparent gray. (**A**) Aromatic amino acids in different groups are highlighted by different colors. Red, yellow, green, blue, and cyan sticks are used to represent aromatic amino acids in Groups 1–5, respectively. (**B**) In different groups, the distances between proximal carbon atoms of aromatic rings are measured. (**C**) The tentative helical pathway for electron transfer in the pilus is represented. Red, green, and yellow spheres highlight the positions of F1, F24, and Y27, respectively. The distances between proximal carbon atoms in neighboring aromatic rings are indicated.

The aromatic rings from the optimized structure have a compact geometry that may provide an electron transfer pathway (Figure 5). The distances between proximal carbon atoms of aromatic rings were measured with the measurement module in Pymol [45]. The distances between aromatic amino acids are shown in Figure 5. The spatial distribution of aromatic amino acids (Figure 5A) and the distances between aromatic amino acids (Figure 5B), showed that the 16 aromatic amino acids of *G. uraniireducens* pilins from different monomers were clustered into five groups. Namely, Group 1 (F1 + F24 + Y27, red sticks in Figure 5A), Group 2 (F32 + Y129 + F142 + Y152 + F156, yellow sticks in Figure 5A), Group 3 (W54 + W75 + W190, green sticks in Figure 5A), Group 4 (Y51 + Y57, blue sticks in Figure 5A), and Group 5 (Y167 + F173 + Y184, cyan sticks in Figure 5A). In each of the five groups, the aromatic amino acids from different monomers stacked with each other and exhibited a helical distribution in the pilus (Figure 5). In the five groups, five potential pathways were found by the shortest path method (Dijkstra’s algorithm, 16 aromatic amino acids in one helical symmetry unit and 16 aromatic amino acids in a neighboring unit were set as the start and the end points, respectively) [46,47]. The five shortest pathways were F1 → F24 → Y27 → F1 (Group 1), F32 → Y129 → Y152 → F142 → F32 (Group 2), W54 → W190 → W75 → W54 (Group 3), Y51 → Y57 → Y51 (Group 4), and Y184 → Y167 → F173 → Y184 (Group 5) (Figure 5B). As shown in Figure 5B, the average distance between the aromatic amino acids was smallest in Group 1. Distances between these residues, i.e., d(F1, F24), d(F24, Y27), and d(Y27, F1) were 3.8, 3.9, and 11.1 Å, respectively (Figure 5C). This result indicated that three aromatic amino acids, i.e., F1, F24 and Y27, from different monomers were packed more tightly than other aromatic amino acids in the pilus. The smaller distance between the aromatic amino acids contributed to the electron transfer efficiency in the pili of *Geobacter* [1,33,47]. The aromatic amino acids F1, F24 and Y27, from different monomers may be expected to provide a tentative helical pathway for electron transfer (Figure 5B,C). In the helically symmetric assembly, F24 and Y27 from a reference monomer P were closely aligned with F1 from monomers P + 2 and P + 3, to form a continuous chain of aromatic rings (Figure 5C). 

**Figure 6:**
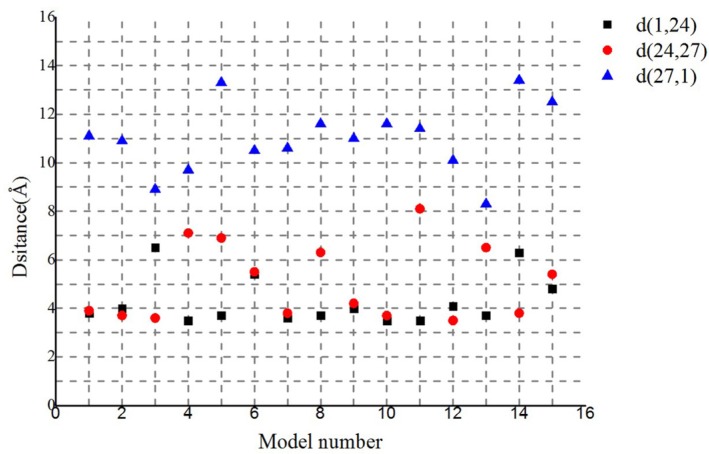
Distances between proximal carbon atoms in neighboring aromatic rings for the 15 lowest energy structures in the largest cluster. As shown in legends, different color points indicate distances between different residues.

Similar chains of aromatic rings (F1 − F24 − Y27 − F1) also appeared in another 14 of the lowest energy structures in the largest cluster (Figure 6). The distances between proximal carbon atoms in neighboring aromatic rings were also measured, i.e., d(F1, F24), d(F24, Y27), and d(Y27, F1) with values of 4, 5 and 11 Å, respectively (Figure 5C or Figure 6). The larger the distance between the aromatic amino acids, the higher the energy barrier that needs to be overcome to achieve electron transfer [26]. The distance 11 Å is too large for the electron transport supported by the mechanism of pi-pi interaction. However, the distance is enough for the mechanism of electron hopping along *G. uraniireducens* pilus [44,48]. The measured distances may support multistep hopping mechanisms for *G. uraniireducens* pilus, but the rate of such electron transfer is expected to be low [32], because these aromatic residues cannot pack as tightly as those in the *G. sulfurreducens* pilus [1,47]. Thus, the mechanism for the electron transfer is likely multistep hoping, and the highest energy barrier exists between the aromatic amino acids Y27 and its neighboring F1 because of the distance between them (Figure 6). These distances are much larger than that in *G. sulfurreducens* (around 3.5 Å), where the conductivity is attributed to pi-pi interactions between aromatic amino acids [1,47]. These results explained the structural basis for the low conductivity of the *G. uraniireducens* pili [1,5].

### 2.4. Co-Localization of Aromatic and Charged Residues in the G. uraniireducens Pilus

**Figure 7:**
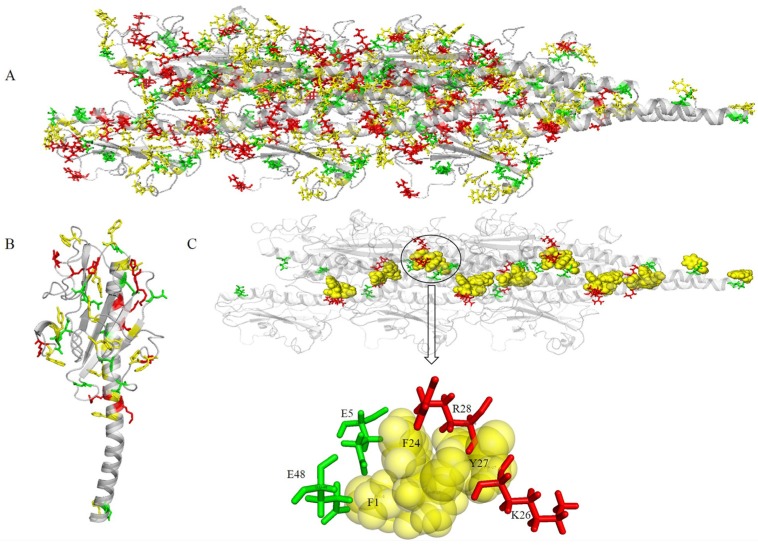
Spatial distribution of aromatics and charged amino acids. Gray schematic represents the structure of a pilus. (**A**) Yellow, red, and green sticks indicate the spatial distribution of aromatic, positive, and negative amino acids, respectively. (**B**) A monomer extracted from structure (**A**). (**C**) Four charged amino acids (E5, K26, R28, and E48) in close proximity to the aromatic amino acids of the charge transport path.

All the aromatic amino acids were in close proximity to charged amino acids (Figure 7A). A monomer was extracted from the predicted structure to help visualize the spatial distribution of aromatic and charged amino acids (Figure 7B). The aromatic amino acids were distributed uniformly in the *G. uraniireducens* pilus. The preferential distribution of charged amino acids was in the core of the pilus (positive charges) or on the surface (globular head domain, negative charges) (Figure 7A,B). Negatively-charged amino acids in the pilins globular head domain contributed greatly to the negative surface potentials (Figure 4B or Figure 7A). The areas of positive potential were small and confined to uncharged regions (Figure 4B or Figure 7A).

Two negatively charged residues (E5 and E48) and two positively charged residues (K26 and R28) were in close proximity to three aromatic amino acids (F1, F24 and Y27), and therefore close to the potential charge transport pathway (Figure 7C). The inter-aromatic distances and the configuration of the aromatic residues involved in the contacts depend on the local electrostatic environment [32], which likely influences the rate of electron transfer through the pili. The local electrostatic environment of aromatic amino acids in the pilus of *Geobacter* is mainly attributed to charged amino acids. The distribution of the aromatic and charged amino acids in the pilus suggested that the four charged amino acids (E5, K26, R28, and E48) may play an important role in the electron transfer process. Furthermore, because nanowires are required to form electrochemically active biofilms in microbial fuel cells, the electrochemical activity of the biofilms depends on the conductivity of the pili as well as its ability to stabilize and support a multilayered biofilm community [33]. The charged surface properties and net charge of the pilus could affect the ability to transfer electrons to redox-active components in the biofilm matrix such as c-cytochromes.

## 3. Materials and Methods

### 3.1. Overall Workflow

**Figure 8:**
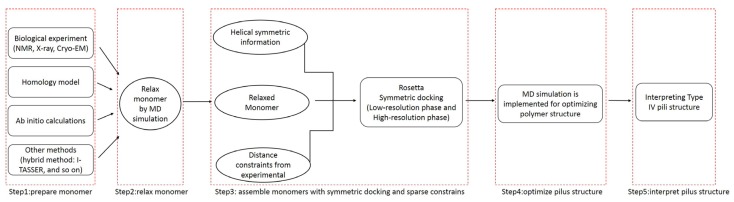
Workflow for Type IV pilus structure modeling and interpretation.

The use of molecular dynamics (MD) simulations to improve the docking process has clear advantages: proteins were simulated in a real biological environment and dynamic effects were considered [31]. The molecular dynamics simulations enabled reasonable model structures of T4Ps, and then allowed interpretation of the functions of T4Ps. As shown in Figure 8, the whole workflow of the proposed method includes five steps.

To obtain a more reasonable pilin conformation, the monomer was refined by a MD simulation [32]. Subsequently, Rosetta was used to obtain the incipient pilus structure by assembling the refined monomers with symmetric docking and sparse constraints. To obtain an appropriate preliminary pilus structure, the assembly involved two stages: low- and high-resolution phases. The low-resolution phase was performed to search for potential pilus structures from a wide sampling space. In the high-resolution phase, side chains were added, and a small initial perturbation and a fast simulated annealing step were implemented to produce full atomic models with structural details. The monomers were considered to be rigid-bodies during the symmetric docking, thus, a MD simulation was applied to further optimize the incipient pilus structure.

### 3.2. Molecular Dynamics Simulation

MD simulations were performed with the Amber12 package [49]. The force field for protein (pilin/pilus) was Amber ff12SB [50]. An appropriate number of Na^+^/Cl^−^ ions were added to maintain the neutrality of all the studied systems. Then, the protein (pilin/pilus) was inserted in a regular octahedral box of a TIP3P water model with at least a 10-Å distance around the pilin. Finally, all the missing hydrogen atoms of the protein (pilin/pilus) were added by the Leap module of the Amber12 software.

The PMEMD program of Amber12 was performed for energy minimization, heating, and equilibration protocol. The energy minimization consisted of two steps: (1) Minimization of all the water molecules and hydrogen atoms with backbone atoms of the protein (pilin/pilus) constraining with a harmonic constraint potential of 20 kcal mol^−1^ Å^−2^; (2) Minimization of the system and allowing all atoms to move freely without restraint. Each energy minimization step was performed with the steepest descent algorithm (50,000 steps) and the conjugate gradient algorithm (20,000 steps) where the non-bonded interaction cutoff was set to be 8 Å. After minimization, a canonical ensemble (NVT) MD was performed for 500 ps with a harmonic restraint weight of 5 kcal mol^−1^ Å^−2^, during which the systems were gradually heated from 0 to 300 K using a Langevin thermostat with a coupling coefficient of 2 ps^−1^. For the equilibration and subsequent production run, the SHAKE algorithm was used on all atoms covalently bonded to a hydrogen atom, allowing for an integration time step of 2 fs. Subsequently, the system was equilibrated at constant volume and temperature (300 K) with a 2 fs time step for 200 ps while restraining all Cα atoms of the structures with a harmonic constraint restraint weight of 2 kcal mol^−1^ Å^−2^. Following this step, a MD simulation run was performed for a time segment (30 ns for pilin, 60 ns for pilus) with no restraints imposed and a PMEMD module in isothermal isobaric (NPT) ensemble at 300 K with a Berendsen barostat with a target pressure of 1 bar and a pressure coupling constant of 1 ps. In the MD simulation, the time steps were set to be 2 fs, and an 8-Å cutoff was applied to treat the nonbonding interactions, the particle mesh Ewald (PME) method was used to handle long-range Coulombic interactions, and periodic boundary conditions were applied to avoid edge effects in the calculations. The coordinate file was saved every 1 ps and the trajectory was analyzed at every 1 ps with the *Cpptraj* module in Amber12 software package [51].

### 3.3. Symmetric Docking with Sparse Constrains

To construct the initial pilus structure, we applied the knowledge that T4Ps have similar intact structures with a right-hand helical symmetry along their assemblies [20]. Helical symmetry was applied by defining a symmetrical conformation space through six degrees of freedom (DOF) of the rigid-body: the rotation around the axis; the translation along the axis; the distance between the axis and the center of mass (COM) of the monomers; and three orientation dimensions of monomers the (*x*, *y*, and *z*). In the symmetric docking by Rosetta, only one master pilin (refined monomer) was accounted for in the calculation and all other monomers were translated from the master through the six DOFs. The symmetry information of the helix could be inferred from an experimentally determined homologous structure. For Type IV pili, the helical symmetry parameters were deduced from the GC pilus (PDB ID: 2HIL).

Proper distance constraints and ambiguous constraints between pairs of amino acids from adjacent monomers were used to narrow down the sampling space. Distance information was obtained from constrained pairs of residues (e.g., F1/E5 of T4P [52], V9/L16 of GC pilin [11]) from known structures and related experimental results such as cysteine crosslinking, salt bridge charge reversal experiments, and hydrogen/deuterium exchange mass spectrometry.

Ambiguous contacts between two residues described above were used when the arrangement of the monomers in Type IV pili was unknown. These ambiguous contacts were delineated as an enumeration of all combinations of the residue pairs, which were from two different monomers, C1, C2, …, C*n*. From min (C1, C2, …, C*n*), the ambiguous constraint was set on pairs that had a minimum from all the energy scores. Here, the total number of monomers was fixed to be 15 (chain names are denoted as consecutive letters: A, B, C, D, …, O). Owing to symmetry, ambiguous contact constraints were only added to the master monomer in the middle and the upper seven monomers. These sparse constraints were applied with Rosetta Constraint Files [27]. Before the symmetric docking, the monomer was aligned along the pilus axis by PyMOL to define the initial position and facilitate the search process. Symmetric docking of monomers with sparse constraints was implemented in Rosetta to obtain 1000 models of full atomic pilus models.

We used a combination of a clustering process (Cut off = 1.75 Å) and energy scores of the first three consecutive monomers (chain names: A, B, and C), to evaluate the models obtained from symmetric docking. The “cluster” application in Rosetta was performed according to structure similarity. The algorithm of cluster definition was based on an old Bradley program (silent_cluster_c) [53]. For the low- and high-resolution models, both the total energy and interface energy of the monomers were applied. We considered interface energy in the high-resolution models because it can approximate binding energy and reflects the stability of pilin assembly. To verify the feasibility of the proposed method, RMSDs were calculated by comparing docking models to the native structure (PDB ID: 2HIL and 5KUA) considering only three consecutive monomers.

### 3.4. Energy Penalty Functions

To use constraint information during the assembly processes, the distance constrains between pairs of atoms were characterized as energy penalty functions for Rosetta. When the Euclidean distance between two atoms was either too small or too large, the function was performed to ensure that models were correctly penalized. A flat harmonic function was applied to fit distance constraints:

(1) $$f\left(d\right)=\{\begin{array}{c} 0,\;\left|d-x_{0}\right|\leq t\\ {\left(\frac{d-x_{0}-t}{sd}\right)}^{2},\;d>x_{0}-t\\ {\left(\frac{d-x_{0}-t}{sd}\right)}^{2},d<x_{0}-t \end{array}$$

where *x*_0_ is an approximation of distance between a pair of atoms, which represents the center of constrains; *sd* denotes standard deviations of Euclidean Distance (*d*) between two atoms; and tolerance (*t*) means the acceptable bound of constraints. These three variables were set to be *x*_0_ = 10, *sd* = 0.5, tolerance = 5, and the constraints were added to the Cα atoms of each residue in the low-resolution phase. In the high-resolution phase, these three variables were set to be 4, 2, and 0.5, respectively, and the constraints were enforced on N-O atom pairs in salt bridges [54].

### 3.5. Electrostatic Properties

Electrostatic interactions are found in almost all biomolecular systems and processes. To help predict binding sites between the pilus and surface appendages, we used a visual inspection of the electrostatic potential at a solvent-accessible surface. The electrostatic properties of the structures were calculated with the Adaptive Poisson–Boltzmann Solver (APBS) and PDB2PQR [55]. High-quality 3D images of pilin/pilus were drawn with PyMOL [45]. The distances between proximal carbon atoms in neighboring aromatic rings were measured with the Measurement module in Pymol. The shortest path method was calculated using the Dijkstra’s algorithm [46]. Command lines for execution of the MD simulations and Symmetric docking are shown in the end of supplemental material.

## 4. Conclusions

Our proposed method represents an improvement because it considered side-chain flexibility, which has been ignored in previous approaches [1,26,27,32,40]. Although the earlier methods of Campos (2011) and Xiao (2015) involved sparse constrains, our predicted structure was closer to the native structure of GC pilus [27,40]. The results showed that the similarity between the native structure and our optimized model was good with an RMSD = 2.308 Å, which is within the resolution of experimental measurements. This improvement may be attributed to the molecular dynamics simulations. Furthermore, the feasibility of the proposed method was also verified by remodeling the pilus structure of *Neisseria meningitidis* (RMSD = 1.972 Å)*.* Hence, the method was applied to investigate the structural basis of conductivity in *G. uraniireducens* pili. The proposed method predicted the structure of *G. uraniireducens* pilus, in which a core chain of aromatic amino acids facilitated electron transport along the pili.

Understanding these structures provides important insights into their biological functions. Clustering of aromatic amino acids (F1, F24 and Y27) in *G. uraniireducens* pilus created paths for electron transport. The distances between neighboring aromatic rings in the potential conductive path were 4, 5 and 11 Å. Although these distances could support multistep hopping, the conductivity implied by the model would be very low. This finding is consistent with multiple lines of experimental evidence that have suggested that *G. uraniireducens* pili have low conductivity (3 × 10^−4^ S/cm) [5,39]. Furthermore, the aromatic amino acids were co-located with charged amino acids in the *G. uraniireducens* pilus model. The local electrostatic environment around the aromatic contacts and the surface properties of the pilus probably influenced the aromatic configuration and promoted efficient electron transfer [32].

The proposed method provided reasonable predictions of the conformation of this conductive biological structure and valuable information for understanding the functions of *Geobacter* pilus. This method may be used to supplement biological experiments and rapidly predict pilus models where X-ray, EM or Cryo-EM data are unavailable. Moreover, the method could also be applied to predict and interpret the structures and details of other complex macromolecules, which assemble with helical symmetry, such as Type II Secretion System pseudopilus [7,10]. Meanwhile, the proposed method has the potential to be applied in DNA-binding protein prediction [56], by finding possible DNA binding sites on the surface of protein such as deep groove. Furthermore, tubule could be reconstructed by the proposed method, and then its structural details could help to detect tubule boundary [57]. Lastly, construction and interpretation of the pilus structure of *Geobacter sulfurreducens PCA/KN400* are the focus of our future work for explaining why the *Geobacter* species may differ substantially in their mechanisms for long-range electron transport.

## Figures and Tables

**Table 1:** Matrix of pilus RMSD values.

	GC1	GC2	GC3	GC4
GC1	0	2.739	3.290	2.308
GC2	-	0	2.386	0.672
GC3	-	-	0	2.343
GC4	-	-	-	0

## Supplementary Materials

The following are available, Figure S1: RMSDs for C-alpha of backbone atoms of the GC pilin, Figure S2: The six degrees of freedom for representing symmetric information of pilus, Figure S3: The landscape of interfacial energy score for GC pili, Figure S4: The values of total energy, temperature, pressure, and volume from trajectory file of 60 ns pilus MD simulation, Figure S5: RMSDs for C-alpha of backbone atoms of the *G. uraniireducens* pilin, Figure S6: The difference between predicted and relaxed structure of *G. uraniireducens* pilin, Figure S7: The landscape of Interface energy, Figure S8: The landscapes of subunits_per_turn, rise, rotation angle and diameter for 15 lowest energy models in the biggest cluster of high-resolution phase , Table S1: Predicted *Neisseria meningitidis* (NM) pilus was closer to the native structure than previous model.
