# Supplementary material for: Predicting and Interpreting the Structure of Type IV Pilus of Electricigens by Molecular Dynamics Simulations
Source: Molecules. 2017 Aug 12;22(8):1342. doi: 10.3390/molecules22081342 (PMC6152092; doi:10.3390/molecules22081342)
Supplement: Supplementary file 1 [file molecules-22-01342-s001.zip › molecules-210605-supplementary.pdf]

Predicting and Interpreting the Structure of Type IV Pilus of Electricigens by Molecular Dynamics Simulations

Chuanjun Shu, Ke Xiao, Changchang Cao, Dewu Ding and Xiao Sun *

State Key Laboratory of Bioelectronics, School of Biological Science and Medical Engineering, Southeast University, Nanjing 210096, China; chuanjunshu@seu.edu.cn (C.S.); kxiao@seu.edu.cn (K.X.); caochch@gmail.com (C.C.); dwding2008@aliyun.com (D.D.)

* Correspondence: xsun@seu.edu.cn; Tel.: +86-025-83795174

Supporting information

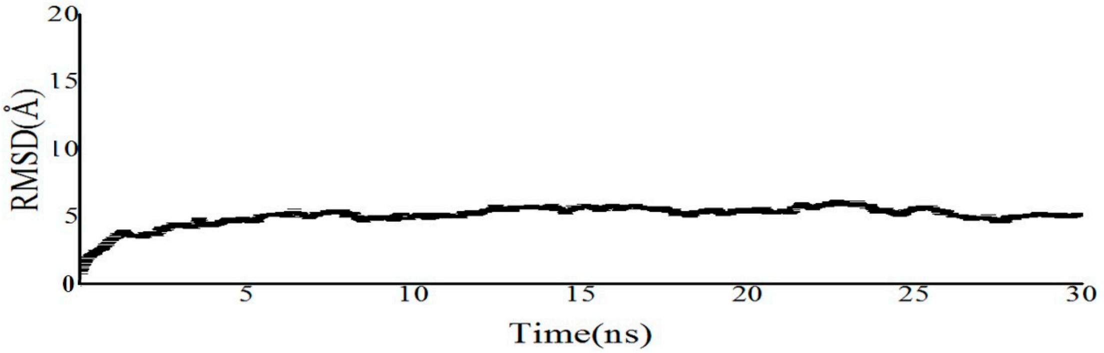

Figure S1. RMSDs for C-alpha of backbone atoms of the GC pilin.

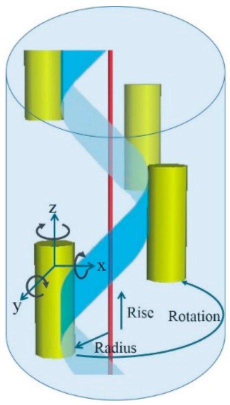

Figure S2. The six degrees of freedom for representing symmetric information of pilus. The light blue and yellow cylinder indicate the sketch of pilus and monomer, respectively. x, *y*, and *z* represent three dimensions of orientation of monomers. The other three degrees of freedom are the rotation around the axis, the translation along the axis and the distance between the axis and the center of mass (COM) of monomers.

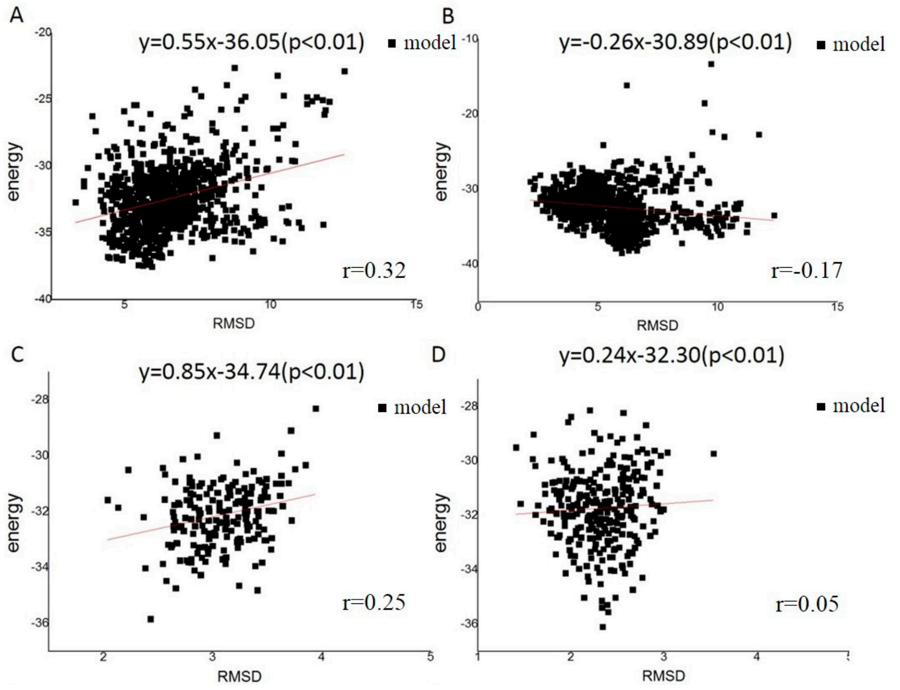

Figure S3. The landscape of interfacial energy score for GC pili. The black dots show the distribution of RMSDs for the models. The red liner and formula indicate the correlation between RMSD and the energy. Figure A and B show all the models of low-resolution phase that symmetric docking respectively from the relaxed monomer and NMR monomer. The largest cluster of A and B are described in C and D. The result indicate that relaxed monomer cause positive correlation between structural energy scores and RMSD values.

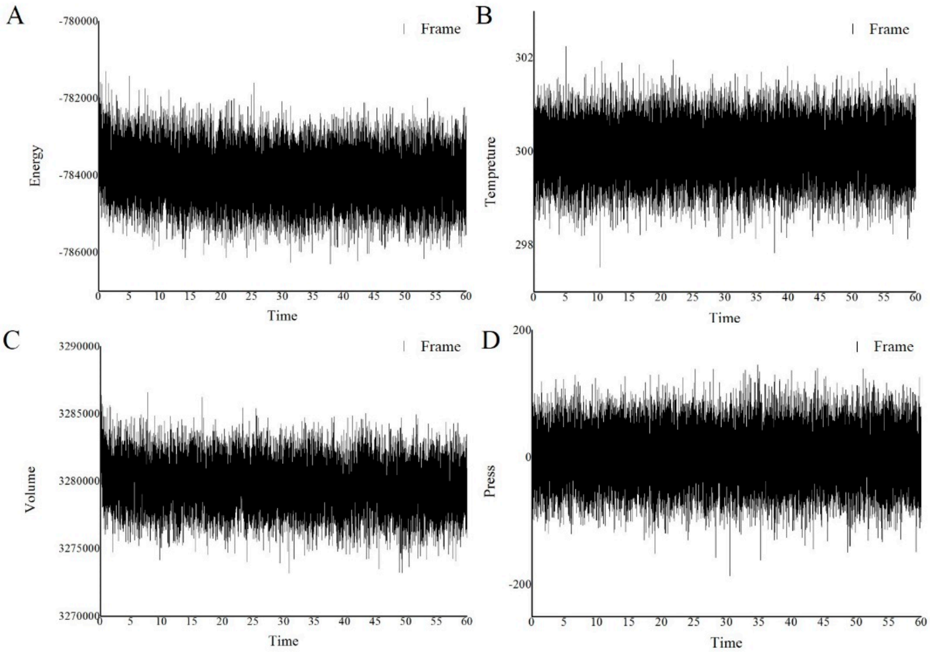

Figure S4. The values of total energy, temperature, pressure, and volume from trajectory file of 60ns pilus MD simulation.

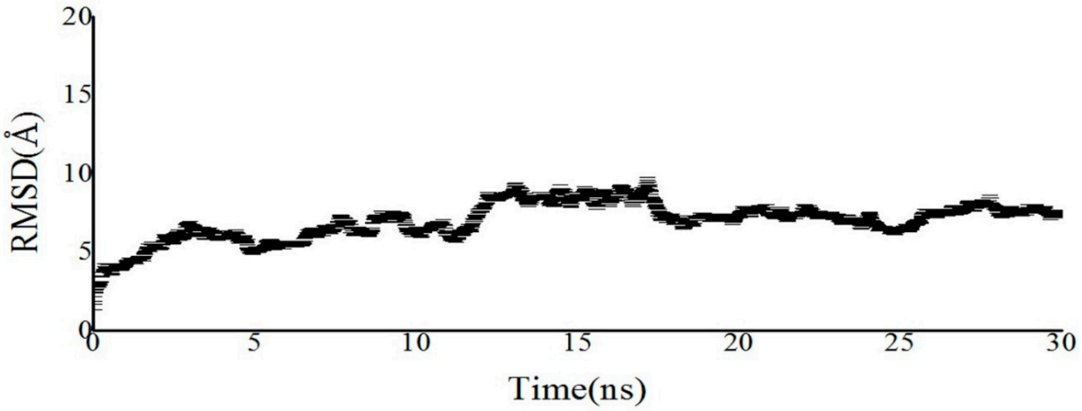

Figure S5. RMSDs for C-alpha of backbone atoms of the *G. uraniireducens* pilin. Three Na+ was added to electro neutralize the simulated system. 30 ns MD simulations were carried out with a PMEMD model in Amber12 software

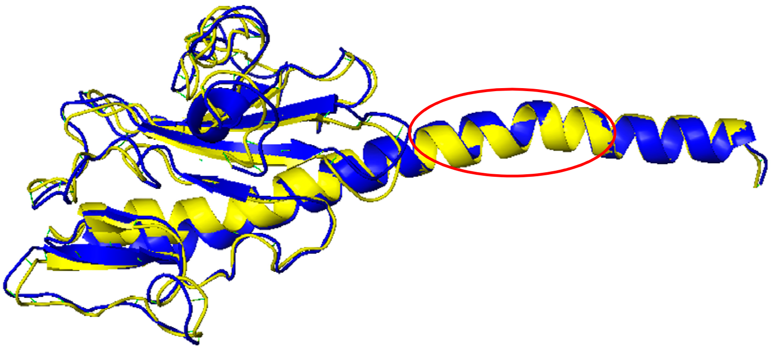

Figure S6. The difference between predicted and relaxed structure of *G. uraniireducens* pilin. The blue and yellow represent the predicted and relaxed structure, respectively. The result show that optimization of monomer leaded to a decrease in the degree of curvature for alpha-helix region.

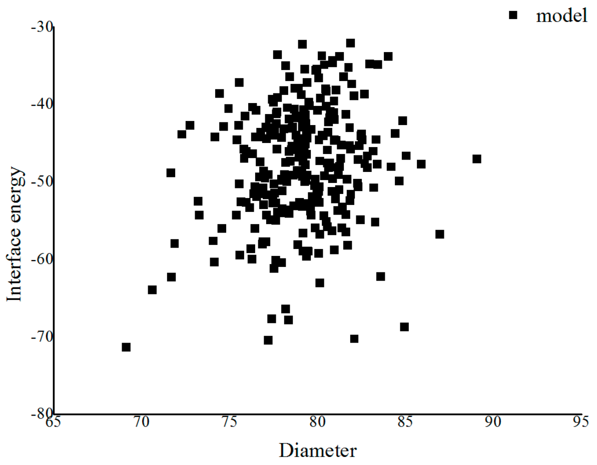

Figure S7. The landscape of Interface energy. The aromatic amino acids and charged amino acids are set as constraint pairs. The cut off is set as 4. There are 100 clusters. The biggest cluster has 277 models.

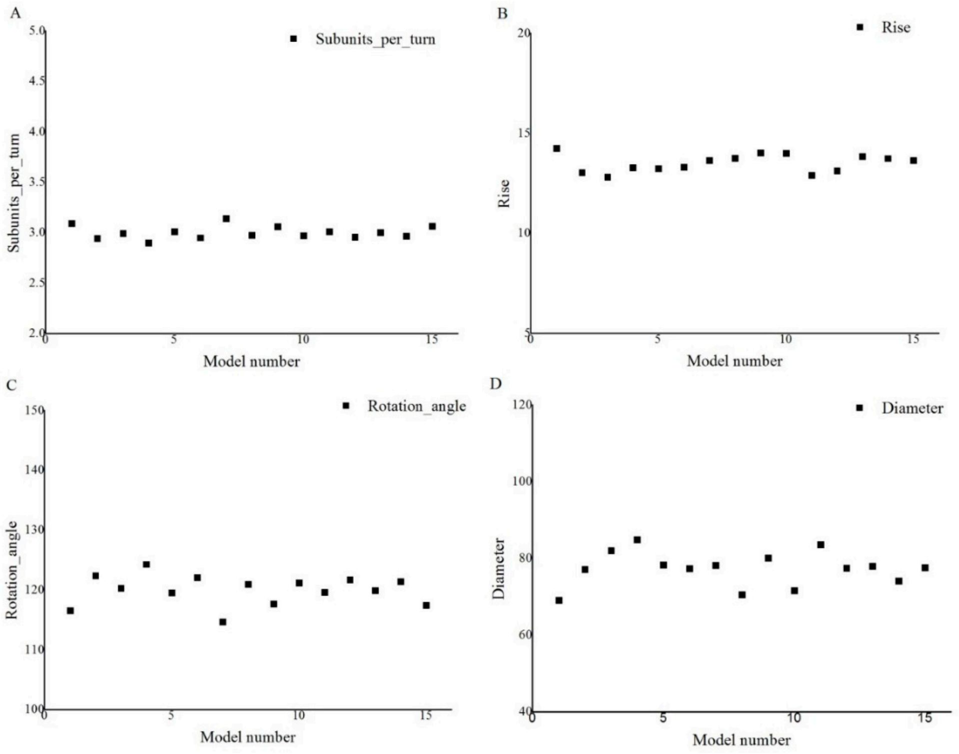

Figure S8. The landscapes of subunits_per_turn, rise, rotation angle and diameter for 15 lowest energy models in the biggest cluster of high-resolution phase.

Table S1. Predicted *Neisseria meningitidis* (NM) pilus was closer to the native structure than previous model.

	NM1	NM2	NM3	NM4
NM1	0	2.376	3.292	1.972
NM2	-	0	4.324	2.855
NM3	-	-	0	3.992
NM4	-	-	-	0

NM1, NM2, NM3 and NM4 represent native structure (PDB ID: 5KUA), pilus structure obtained from symmetric docking refined monomer, pilus structure obtained from previous method, and the optimized pilus structure, respectively. Data shown in the table were obtained by quantifying the conformational changes between the two structures (units are Å). The initial monomer was extracted from the structure of pilus (PDB ID: 5KUA). The details of the helical symmetry parameters were set as follows: (1) Rise (Å) was set to be 5–15, (2) the rotation_angel (°) was set to be 80–130, (3) the radius_to_com (Å) was set to be 15– 30, and (4) the number of monomers was set to be 15. The charged amino acids were set to be constrained pairs.

Command lines for analysis of MD's production:

RMSD.ptraj :
parm practice.top

```
trajin practice.mdcrd
rms @CA out rmsd_first.dat first
run
$: cpptraj –i RMSD.ptraj > RMSD_ptraj.out
```

The symmetric definition files could be obtained from GC pilus structure (PDB ID: 2HIL) by running the make_symmdef_file.pl script:

```
>perl $rosettadir/rosetta_source/src/apps/public/symmetry/make_symmdef_file.pl -m HELIX -p 2HIL.pdb -r 20.0 -a A -b B -t 7 > symm.def
```

Additional degrees of freedom (DOF) would be set to make sure the sampling is taken under specific situations, i.e. specific initial positions and searching ranges.

Command lines for execution of the low- and high-resolution steps:

Step 1: Low-resolution phase

A pdb file (refined.pdb) containing the monomeric structure, relaxed and aligned along the helical axis, is used as an input. Constraint file lowres.cst is generated by hand according to the specific constraint pairs.

Execution of the Rosetta procedure:

```
>$rosettadir/main/source/bin/SymDock.linuxgccrelease \
-in:file:s refined.pdb \
-database $rosettadir/main/database \
-symmetry:symmetry_definition symm.def \
-packing:ex1 \
-packing:ex2aro \
-out:nstruct 3000 \
-out:pdb \
-symmetry:initialize_rigid_body_dofs \
-constraints:cst_file lowres.cst \
-constraints:cst_weight 10.0 \
-docking:dock_lowres_filter 1.0 1.0 1.0 \
-mute core
```

Step 2: High-resolution phase

The low-energy region from step 1 is taken as the starting range for the high-resolution phase. The symmetric definition file symm_highres.def is generated from 2HIL, using the same Perl script mentioned above. Constraint file highres.cst is generated by hand according to the specific constraint pairs.

Execution of the Rosetta procedure

```
>$rosettadir/main/source/bin/SymDock.linuxgccrelease \
-in:file:s refined.pdb \
-database $rosettadir/main/database \
```

```
-symmetry:symmetry_definition symm_h.def \
-packing:ex1 \
-packing:ex2aro \
-out:nstruct 1000 \
-out:file:fullatom \
-symmetry:initialize_rigid_body_dofs \
-constraints:cst_file lowres.cst \
-constraints:cst_weight 10.0 \
-constraints:cst_fa_file highres.cst \
-constraints:cst_fa_weight 100.0 \
-docking:dock_lowres_filter 1.0 1.0 1.0 \
-mute core \
-docking:kick_relax \
-docking:dock_ppk
```
